# Application of Minocycline-Containing Bismuth Quadruple Therapies as First-Line Regimens in the Treatment of *Helicobacter pylori*

**DOI:** 10.1155/2019/9251879

**Published:** 2019-12-18

**Authors:** Lingyun Zhang, Yu Lan, Qi Wang, Yuexia Zhang, Xiaobei Si

**Affiliations:** Department of Gastroenterology, Beijing Jishuitan Hospital. NO 68, Hui Nan Road, Changping District, Beijing 100096, China

## Abstract

**Aim:**

To evaluate the eradication rate, safety, and compliance of minocycline-containing bismuth quadruple regimens in patients with an untreated *Helicobacter pylori* (*H. pylori*) infection.

**Methods:**

A total of 360 patients with an untreated *H. pylori* infection were enrolled in this study between June 2017 and October 2018. Patients were randomly divided into a minocycline/amoxicillin (RMAB) group, a minocycline/metronidazole (RMMB) group, or an amoxicillin/clarithromycin (RACB) group, and all groups received a combined treatment approach with rabeprazole and bismuth to create a quadruple regimen for 14 days. A 3 to 5-day follow-up was adopted to evaluate the safety and compliance of medications after medicine administration. ^13^C-urea breath test was performed to confirm the eradication of *H. pylori* 4-12 weeks after therapy.

**Results:**

No significant differences were observed at baseline data among the three groups (*p* > 0.05). Based on the intent-to-treat analysis, the eradication rates of the RMAB group, RMMB group, and RACB control group were 85.7% (102/119), 77.1% (91/118), and 71.7% (86/120), respectively, with significant difference (*χ*^2^ = 7.015, *p* = 0.030). According to per protocol analysis, the eradication rates of RMAB group, RMMB group, and RACB group were 89.5% (102/114), 84.3% (91/108), and 76.8% (86/112), respectively, with statistically significant differences (*χ*^2^ = 6.673, *p* = 0.036). The eradication rates of the RMAB group and RACB group were significantly different (*p* < 0.05). The overall incidences of adverse events in the three groups were 30.0%, 37.5%, and 40.0%, respectively (*p* > 0.05). Nausea, epigastric discomfort, and dizziness were more obvious in patients in the RMMB group compared to the other two groups (*p* < 0.05). Moreover, two patients discontinued due to severe dizziness and nausea in the RMMB group. A taste disorder was more prominent in patients in the RACB group compared to patients in the other two groups (*p* < 0.05), and one patient discontinued because of the bitterness in the mouth. Soon after discontinuation of the medicine, all adverse events disappeared*. Conclusion.* The bismuth quadruple regimen using minocycline/amoxicillin showed a better eradication effect with fewer side effects in patients with untreated *H. pylori* infections. The bismuth quadruple regimen with minocycline/metronidazole had a good eradication effect with more obvious side effects and might be recommended to patients with penicillin allergy.

## 1. Introduction

*Helicobacter pylori* (*H. pylori*) infection is a global disease, which has attracted increased attention due to its correlation with diseases, including peptic ulcer, gastric cancer, mucosa-associated lymphoid tissue (MALT) lymphoma, and other intra- and extragastric diseases [1]. However, in recent years, due to the increase in resistance of *H. pylori* to various antibiotics, it is increasingly challenging to be eradicated. At present, since the eradication rate of *H. pylori* by the standard triple regimen is significantly reduced, its empirical applications are no longer recommended [2]. The latest domestic and international consensus [1, 2] recommends using the bismuth quadruple regimen as the first-line treatment. However, China faces a more serious problem of antibiotic resistance of *H. pylori*: the primary resistance rates of nitroimidazole, fluoroquinolone, and macrolide antibiotics are all high. In addition, tetracycline and furazolidone show more severe side effects and cannot be obtained in most hospitals in China. Although *H. pylori* is sensitive to the clinically available amoxicillin [3], potent antibiotics that can be combined with amoxicillin to create an eradication regimen are lacking. Minocycline is a semisynthetic tetracycline with fewer side effects. *In vitro* [4] susceptibility studies have shown that similar to tetracycline, *H. pylori* is sensitive to minocycline. However, systematic evaluation of minocycline in clinical eradication therapy is not yet available. In this study, we proposed to evaluate the eradication rate, safety, and compliance of a minocycline-based bismuth quadruple regimen (respectively combined with amoxicillin and metronidazole) in patients with an untreated *H. pylori* infection so as to evaluate the application value of minocycline in *H. pylori* eradication therapy.

## 2. Subjects and Methods

### 2.1. Study Subjects

Patients admitted to Beijing Jishuitan Hospital (Beijing, China) between June 2017 and October 2018 with an untreated *H. pylori* infection were selected for this study. The inclusion criteria were the following: (1) positive for either the ^13^C-urea breath test (^13^C-UBT) or the gastric mucosal tissue rapid urease test and pathological section staining, (2) no history of treatment with *H. pylori* eradication, (3) aged 18-75 years old, and (4) fully informed and agreed to participate in this study. The exclusion criteria were the following: (1) received medications that might affect the study results up to 4 weeks prior to enrollment, including proton pump inhibitors (PPIs), bismuth, or antibiotics; (2) patients with malignant tumors; (3) patients with a history of upper gastrointestinal surgery; (4) patients with systemic diseases, including severe liver or kidney damage; (5) patients allergic to the medicines used in this study; (6) women who were pregnant or lactating; (7) patients with long-term use of glucocorticoids or nonsteroidal anti-inflammatory medicines; and (8) patients with mental abnormalities who had difficulties cooperating with the investigator. This study was approved by the ethics committee of the Beijing Jishuitan Hospital (Beijing, China).

### 2.2. Grouping and Treatment Regimens

Of all enrolled patients, demographic information was recorded, and patients were randomly divided into three groups according to the random number table. Specific medicines of the regimens were as follows: minocycline/amoxicillin group (rabeprazole, minocycline, amoxicillin, and bismuth (RMAB) group): rabeprazole (Eisai China Pharmaceutical Co., Ltd., 10 mg, BID)/minocycline (Wyeth-Palace Pharmaceutical Co., Ltd, 1.0 g, BID)/amoxicillin (Kunming Baker Norton Pharmaceutical Co., Ltd., 1.0 g, BID)/bismuth potassium citrate (Livzon Pharmaceutical Co., Ltd., 220 mg, BID); minocycline/metronidazole group (rabeprazole, minocycline, metronidazole, and bismuth (RMMB) group): rabeprazole (Eisai China Pharmaceutical Co., Ltd., 10 mg, BID)/minocycline (Wyeth-Palace Pharmaceutical Co., Ltd, 1.0 g, BID)/metronidazole (Shanxi Yabao Pharmaceutical Co., Ltd., 0.4 g, TID)/bismuth potassium citrate (Livzon Pharmaceutical Co., Ltd., 220 mg, BID); and amoxicillin/clarithromycin group (rabeprazole, amoxicillin, clarithromycin, and bismuth (RACB) group): rabeprazole (Eisai China Pharmaceutical Co., Ltd., 10 mg, BID)/amoxicillin (Kunming Baker Norton Pharmaceutical Co., Ltd., 1.0 g, BID)/clarithromycin (Shanghai Abbott Laboratories, 500 mg, BID)/bismuth potassium citrate (Livzon Pharmaceutical Co., Ltd., 220 mg, BID). All regimens lasted for 14 days.

### 2.3. Evaluation of Efficacy and Safety

The adverse events and compliance of patients were followed 3 to 7 days after the therapy, and patients underwent a ^13^C-UBT 8 to 12 weeks after the therapy. If the result of the test was negative, *H. pylori* was considered successfully eradicated. *H. pylori* eradication rate calculations were assessed by intent-to-treat (ITT) analysis and per protocol (PP) analysis, respectively. Compliance was calculated as the percentage of the number of tablets that were actually taken by the patient to the number of tablets that should have been taken. Compliance greater than or equal to 90% was considered good, and compliance less than 80% was considered poor and was therefore not included in the PP analysis [5].

### 2.4. Statistical Analysis

For the statistical analysis of this study, SPSS (version 19, SPSS Inc., Chicago, IL, USA) was employed. The comparison of measurement data was analyzed by one-way ANOVA. Count data was analyzed by Pearson's *χ*^2^ or Fisher's exact test. The main observation targets included the eradication rate, rate of adverse events, and compliance of patients in three groups. *p* < 0.05 was considered statistically significant.

## 3. Results

### 3.1. Baseline Demographic Data

**Figure 1:**
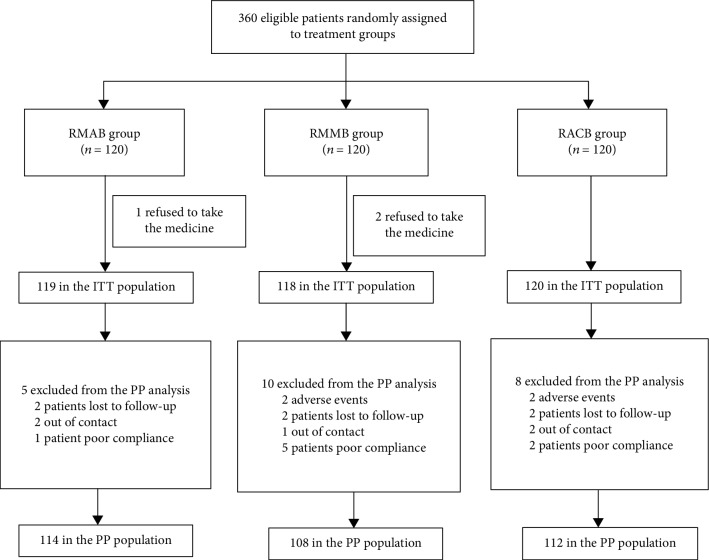
Flow diagram of this study. RMAB group: rabeprazole plus minocycline, amoxicillin, and bismuth; RMMB group: rabeprazole plus minocycline, metronidazole, and bismuth; RACB group: rabeprazole plus amoxicillin, clarithromycin, and bismuth. ITT: intention-to-treat; PP: per protocol.

A total of 360 patients with an untreated *H. pylori* infection were enrolled in this study and were randomly assigned to three groups, with 120 cases per group. Except for the 3 patients who refused to take the medicine, 357 patients were included in the ITT analysis. There were 119 patients in the RMAB group, 118 patients in the RMMB group, and 120 patients in the RACB control group. During the study, a total of 23 patients (6.4%) were not included in the PP analysis due to adverse events, loss of contact, poor compliance, or refusal to reexamination, including 5 patients in the RMAB group (4.2%), 10 patients in the RMMB group (8.5%), and 8 cases (6.7%) in the RACB group (Figure 1). Baseline demographic data of patients in the three groups were well matched (Table 1).

### 3.2. Eradication Rate

According to ITT analysis, the eradication rates of the RMAB group, RMMB group, and RACB control group were 85.7% (102/119), 77.1% (91/118), and 71.7% (86/120), respectively. Moreover, the differences observed were statistically significant (*χ*^2^ = 7.015, *p* = 0.030). Based on PP analysis, the eradication rates of the RMAB group, RMMB group, and RACB control group were 89.5% (102/114), 84.3% (91/108), and 76.8% (86/112), respectively. The differences were statistically significant (*χ*^2^ = 6.673, *p* = 0.036) (Table 2). The eradication rate of RMAB group was significantly higher compared to that of RABC group, and the difference was statistically significant (*p* = 0.008 for ITT analysis; *p* = 0.011 for PP analysis). No statistical differences were found among other groups.

### 3.3. Safety and Compliance

No significant differences were observed in the overall incidence of adverse events among the three groups (Table 3). Melena was observed in all three groups. Furthermore, the adverse events of patients in the RMAB group mainly included dizziness and digestive tract discomfort, and overall, the symptoms were mild. No patients discontinued the medicine due to adverse events, and the symptoms disappeared quickly after termination of treatment. The adverse events of patients in the RMMB group mainly included dizziness and upper gastrointestinal discomfort, with a significantly higher incidence compared with the other two groups (*p* < 0.05). Moreover, 2 patients discontinued the medicine due to obvious dizziness and nausea. The adverse events were fully relieved after discontinuation of the medicine. In the RACB control group, the adverse events mainly included the taste disorder, which was more prominent than in the other two groups (*p* < 0.05). One patient discontinued due to the bitterness in the mouth, and one patient discontinued due to a rash which was possible allergic reactions. Other adverse events were mild and were completely relieved after discontinuation of the medicine.

In this study, a total of 15 patients had a medicine compliance of less than 90%, and 95.8% (342/357) of patients presented good compliance, which were 96.6% (115/119) in the RAMB group, 94.9% (112/118) in the RMMB group, and 95.8% (115/120) in the RACB group, respectively. No significant differences in medicine compliance were observed among the three groups (*p* > 0.05).

## 4. Discussion

The results of the study indicated that the eradication effect of the RMAB Group was significantly better than that of the RACB control group, without an increase in the incidence of adverse events, and could be recommended as a first-line treatment regimen for patients with an *H. pylori* infection. The eradication rate of the RMMB was not as good as that of the RMAB group but was higher than the RACB control group (without statistically significant). The RMMB group exhibited obvious dizziness and digestive tract discomfort, but all of the adverse reactions relieved after discontinuation of the medicine. RMMB might be recommended to patients with an *H. pylori* infection who are allergic to penicillin.

In recent years, the antibiotic resistance of *H. pylori* has increasingly become more serious [2]. In Europe, the resistance rate of clarithromycin and metronidazole was 17.5% and 34.9%, respectively [6]. But in China [3], it has been shown that the resistance rates of clarithromycin, metronidazole, and levofloxacin to *H. pylori* were 52.6%, 63.4% and 54.8%, respectively, higher than in Europe. Multidrug resistance was also commonly found in the *H. pylori* strains. In Beijing [4], 26.5% of the *H. pylori* strains were resistant to both clarithromycin and metronidazole, and 22.7% strains were resistant to clarithromycin, levofloxacin, and metronidazole. This high resistance has severely affected the eradication effect of many therapies including triple therapy, sequential therapy, concomitant therapy, and even mixed therapy [7]. It is hard to empirically choose the ideal eradication regimens. In China, the most recent consensus [1] recommends the use of amoxicillin or a tetracycline-based bismuth quadruple regimen as the first-line treatment. But tetracycline could not be obtained, and no ideal antibiotics can be combined with amoxicillin to form a bismuth quadruple regimen. For patients allergic to penicillin, no effective therapeutic medicine is available. Therefore, it is urgent to explore other medicine and regimen for *H. pylori eradication.*

Minocycline is a semisynthetic tetracycline that is synthesized by reintroducing the dimethyl nitrogen group at the 4-position of the tetracycline core—the hydrogenated tetraphenylene—to the 7-position. Its mechanism of action is similar to that of tetracycline. It specifically binds to the 30S subunit of the bacterial ribosome at the A position to prevent the binding of aminoacyl-tRNA at this position, so as to prevent peptide chain extension and bacterial protein synthesis. Minocycline is the most potent semisynthetic tetracycline and has a higher safety profile compared to tetracycline as well as a longer half-life [8]. It could be used twice a day and has good patient compliance. Previous in vitro drug sensitivity studies have demonstrated that, similar to tetracycline, *H. pylori* has a low resistance to minocycline [4, 9]. In clinical trials, certain cases of the successful elimination of multiple resistant *H. pylori* infection by a triple regimen with minocycline selected based on drug sensitivity were reported in Japan [10–12]. However, few systematic clinical studies on the application of minocycline in *H. pylori* infection have been published, and applications were mainly focused on retreatment of patients with high resistance [13, 14]. The number of patients enrolled in these studies was small, and the treatment duration was short; therefore, the significance was limited. In our previous study, we evaluated the efficacy of the minocycline/amoxicillin bismuth quadruple regimen in patients who were retreated with *H. pylori* treatment and showed that the eradication rate was 79.4% (ITT analysis) and 84.1% (PP analysis), with a good efficacy and safety profile [15, 16]. In this study, the results demonstrated that the effect of bismuth quadruple regimens with minocycline had a good effect of *H. pylori* eradication, especially the regimen of minocycline combined with amoxicillin.

The classical quadruple regimen using tetracycline and metronidazole has been highly regarded [2], especially in patients who are allergic to penicillin. However, application of this regimen is limited due to the unavailability of tetracycline and the high resistance of metronidazole. In recent studies, it has been shown that the resistance rate of metronidazole was relatively stable for several years, and increasing the dose and frequency can some extent overcome its resistance [2, 17]. Therefore, in this study, we intended to replace tetracycline with minocycline and combine it with metronidazole to create a novel classic quadruple regimen. Considering with the adverse reaction of metronidazole, we used the dose of 1.2 g/d, but not 1.6 g/d, which was recommended by the Fifth Chinese National Consensus Report on the management of *H. pylori* infection [1]. The results indicated that the clinical efficacy was slightly better compared to the RACB group and could serve as a recommended treatment for patients who were allergic to penicillin.

Based on the results of this study, the total incidence of adverse events was comparable among the three groups. Obvious melena was observed in patients in all three groups, which was mainly caused by bismuth. The two groups receiving a minocycline-containing bismuth quadruple regimen exhibited obvious dizziness, which might mainly be associated with the reversible vestibular response of minocycline (characterized by dizziness, tinnitus, ataxia with nausea, vomiting, etc.). Vestibular dysfunctions often occurred at initial administration, and most patients recovered 24 to 48 hours after discontinuation of the medicine. Dizziness in patients treated by RMAB group was significantly less pronounced when compared to the RMMB group. These findings were considered to be associated with adverse events caused by metronidazole. The main adverse events of metronidazole included digestive discomfort and nervous system symptoms, including dizziness and headache. When combined with minocycline, the frequency of dizziness and headaches was higher, and the degree was more severe. Some patients could no longer tolerate this and discontinued in the middle of the study. The adverse events of the medicine had a certain impact on its clinical application, which limited the research direction of further increasing the dose of metronidazole to improve the eradication rate. However, for patients who are allergic to penicillin, the eradication rate of the regimen is acceptable. Therefore, this regimen might be considered with close monitoring of adverse events.

## 5. Conclusions

In summary, our study suggests that the bismuth quadruple regimen containing minocycline/amoxicillin has a better eradication effect and less adverse effect in patients with an untreated *H. pylori* infection. The bismuth quadruple regimen with minocycline/metronidazole has an acceptable eradication effect but more adverse events, so might be recommended to patients with an *H. pylori* infection who are allergic to penicillin. However, this study was a single-center clinical study, and no *H. pylori* culture and drug susceptibility tests were conducted. Multicenter clinical studies with increased sample sizes are required to confirm the value of minocycline in patients with an *H. pylori* infection.

## Figures and Tables

**Table 1:** The baseline demographic data.

Parameter	Value for the group			*p*
	RMAB (*N* = 120)	RMMB (*N* = 120)	RACB (*N* = 120)	
Age (yr (range))	44.9 ± 13.99 (23~74)	40.8 ± 13.80 (18~70)	40.9 ± 13.30 (23~75)	0.551
Gender (male/female)	53/67	62/58	52/68	0.362
Smoking status (*n*, %)	10 (8.3)	13 (10.8)	13 (10.8)	0.757
BMI (kg/m^2^)	23.9 ± 3.65	23.1 ± 3.33	23.2 ± 4.47	0.331
Peptic ulcer history (*n*, %)	22 (18.3)	32 (26.7)	23 (19.2)	0.222

RMAB group: rabeprazole plus minocycline, amoxicillin, and bismuth; RMMB group: rabeprazole plus minocycline, metronidazole, and bismuth; RACB group: rabeprazole plus amoxicillin, clarithromycin, and bismuth. BMI: body mass index.

**Table 2:** *H. pylori* eradication rates of each group in PP and ITT analysis.

Population	Eradication by group (%)						*p*
	RMAB		RMMB		RACB		
	Rate *n*/*N* (%)	95% CI	Rate *n*/*N* (%)	95% CI	Rate *n*/*N* (%)	95% CI	
ITT	102/119 (85.7)	79.3~92.1	91/118 (77.1)	69.4~84.8	86/120 (71.7)	63.5~79.8	0.030^∗^
PP	102/114 (89.5)	83.8~95.2	91/108 (84.3)	77.3~91.2	86/112 (76.8)	68.8~84.7	0.036^∗∗^

^∗^ *p* = 0.008, RMAB vs. RACB; ^∗^*p* = 0.089, RMAB vs. RMMB; ^∗^*p* = 0.335, RMMB vs. RACB. ^∗∗^*p* = 0.011, RMAB vs. RACB; ^∗∗^*p* = 0.250, RMAB vs. RMMB; ^∗∗^*p* = 0.163, RMMB vs. RACB. RMAB group: rabeprazole plus minocycline, amoxicillin, and bismuth; RMMB group: rabeprazole plus minocycline, metronidazole, and bismuth; RACB group: rabeprazole plus amoxicillin, clarithromycin, and bismuth. ITT: intention-to-treatment; PP: per-protocol; CI: confidence interval.

**Table 3:** Side effects of different treatment regiments.

	RMAB	RMMB	RACB	P1	P2	P3
Side effects of treatment, *n* (%)	36 (30.0)	45 (37.5)	48 (40.0)	0.219	0.104	0.691
Nausea	8 (6.7)	18 (15.0)	3 (2.5)	0.038	0.123	0.001
Vomiting	1 (0.8)	2 (1.7)	1 (0.8)	—	1.000	1.000
Epigastric discomfort	13 (10.8)	24 (20.0)	12 (10.0)	0.049	0.833	0.030
Dizziness	8 (6.7)	24 (20.0)	3 (2.5)	0.002	0.123	0.000
Insomnia	3 (2.5)	5 (4.2)	3 (2.5)	0.472	1.000	0.722
Diarrhea	10 (8.3)	9 (7.5)	16 (13.3)	0.811	0.213	0.139
Darkened stool	30 (25.0)	29 (24.2)	33 (27.5)	0.811	0.660	0.555
Taste disorder	3 (2.5)	4 (3.3)	41 (34.2)	1.0000	0.000	0.000
Skin rash	0	0	1			
Tinnitus	0	0	1			
Arthralgia	0	1	0			

P1: RMAB vs. RMMB; P2: RMAB vs. RACB; P3: RMMB vs. RACB. RMAB group: rabeprazole plus minocycline, amoxicillin, and bismuth; RMMB group: rabeprazole plus minocycline, metronidazole, and bismuth; RACB group: rabeprazole plus amoxicillin, clarithromycin, and bismuth.

## Data Availability

The data used to support the findings of this study are available from the corresponding author upon request.
