# Corrigendum to “Application of Minocycline-Containing Bismuth Quadruple Therapies as First-Line Regimens in the Treatment of *Helicobacter pylori*”

**DOI:** 10.1155/2021/9823426

**Published:** 2021-05-18

**Authors:** Lingyun Zhang, Yu Lan, Qi Wang, Yuexia Zhang, Xiaobei Si

**Affiliations:** Department of Gastroenterology, Beijing Jishuitan Hospital, NO 68, Hui Nan Road, Changping District, Beijing 100096, China

In the article titled “Application of Minocycline-Containing Bismuth Quadruple Therapies as First-Line Regimens in the Treatment of Helicobacter pylori” [1], there was an error in Subjects and Methods. This error is shown below:

“(Wyeth-Palace Pharmaceutical Co., Ltd, 1.0g, BID)” should read “(Wyeth-Palace Pharmaceutical Co., Ltd, 0.1 g, BID).”
